# Pre-clinical activity of combined LSD1 and mTORC1 inhibition in *MLL*-translocated acute myeloid leukaemia

**DOI:** 10.1038/s41375-019-0659-6

**Published:** 2019-11-28

**Authors:** Gauri Deb, Bettina Wingelhofer, Fabio M. R. Amaral, Alba Maiques-Diaz, John A. Chadwick, Gary J. Spencer, Emma L. Williams, Hui-Sun Leong, Tamara Maes, Tim C. P. Somervaille

**Affiliations:** 1 Leukaemia Biology Laboratory, Cancer Research UK Manchester Institute, The University of Manchester, Oglesby Cancer Research Centre Building, 555 Wilmslow Road, Manchester, M20 4GJ UK; 2 Computational Biology Support, Cancer Research UK Manchester Institute, The University of Manchester, Oglesby Cancer Research Centre Building, 555 Wilmslow Road, Manchester, M20 4GJ UK; 3 Oryzon Genomics S.A., Carrer Sant Ferran 74, Cornellà de Llobregat, 08940 Barcelona, Spain

**Keywords:** Chemotherapy, Acute myeloid leukaemia

## Abstract

The histone demethylase lysine-specific demethylase 1 (LSD1 or KDM1A) has emerged as a candidate therapeutic target in acute myeloid leukaemia (AML); tranylcypromine-derivative inhibitors induce loss of clonogenic activity and promote differentiation, in particular in the *MLL*-translocated molecular subtype of AML. In AML, the use of drugs in combination often delivers superior clinical activity. To identify genes and cellular pathways that collaborate with LSD1 to maintain the leukaemic phenotype, and which could be targeted by combination therapies, we performed a genome-wide CRISPR-Cas9 dropout screen. We identified multiple components of the amino acid sensing arm of mTORC1 signalling—RRAGA, MLST8, WDR24 and LAMTOR2—as cellular sensitizers to LSD1 inhibition. Knockdown of mTORC1 components, or mTORC1 pharmacologic inhibition, in combination with LSD1 inhibition enhanced differentiation in both cell line and primary cell settings, in vitro and in vivo, and substantially reduced the frequency of clonogenic primary human AML cells in a modelled minimal residual disease setting. Synergistic upregulation of a set of transcription factor genes associated with terminal monocytic lineage differentiation was observed. Thus, dual mTORC1 and LSD1 inhibition represents a candidate combination approach for enhanced differentiation in *MLL*-translocated AML which could be evaluated in early phase clinical trials.

## Introduction

Lysine-specific demethylase 1 (LSD1, also known as KDM1A) has emerged as a candidate therapeutic target in acute myeloid leukaemia (AML), as well as in some solid malignancies [1]. It was initially identified as a core component of an RCOR1 (CoREST) histone deacetylase (HDAC) transcription corepressor complex where it exhibits flavin adenine dinucleotide-dependent demethylase activity vs. mono- and dimethyl-histone H3 lysine 4 (H3K4). Subsequent studies revealed that LSD1 also binds with high affinity to N-terminal sequences of SNAG domain transcription factor family members, an interaction facilitated by molecular mimicry of the histone H3 tail by the SNAG domain [1, 2]. Indeed physical association of LSD1 with the SNAG domain of GFI1 is essential for the activity of GFI1 as a transcription repressor [3].

Pre-clinical studies in AML demonstrated that LSD1 contributes to the differentiation block that is the cardinal feature of the disease: *LSD1* knockdown (KD) or LSD1 pharmacologic inhibition promotes differentiation of, in particular, AML cells with chromosomal translocations targeting *MLL* [4, 5]. Development of more potent and specific tranylcypromine-derivative inhibitors such as GSK2879552 and ORY-1001 [6, 7] has facilitated early phase clinical trials. In AML, ORY-1001 is well tolerated by patients and induces molecular and morphological differentiation of blast cells in leukaemias driven by *MLL* gene rearrangements [8]. Interestingly, LSD1 inhibitors promote differentiation of *MLL* AML cells through disruption of the LSD1/CoREST complex with GFI1 on chromatin; the demethylase activity of LSD1 is not required to sustain the clonogenic activity of leukaemia cells [9].

While early clinical trial results are encouraging, most effective treatments in AML are delivered in combination regimens. Identification of genes and cellular pathways whose loss of function collaborates or synergises with pharmacologic inhibition of LSD1 to promote differentiation represents an attractive strategy for uncovering novel drug combinations for testing in early phase trials. To address this question we used a genome-wide loss-of-function CRISPR-Cas9 screening approach [10].

## Materials and methods

### Human tissue, cell lines, cell culture, reagents and antibodies

Use of human tissue was in compliance with the UK’s Human Tissue Act, 2004. Primary human AML samples were from Manchester Cancer Research Centre’s Tissue Biobank; their use was approved by South Manchester Research Ethics Committee, the Tissue Biobank’s scientific sub-committee, and with the informed consent of the donor. Details of cell lines, culture, reagents and antibodies are in the Supplementary Information.

### Murine experiments

Experiments using NOD-SCID IL2Rγ^−/−^ mice (female, aged 6–12 weeks; Envigo, Shardlow, UK) were approved by Cancer Research UK Manchester Institute’s Animal Ethics Committee and performed under a project license issued by the United Kingdom Home Office, in keeping with the Home Office Animal Scientific Procedures Act, 1986. Dosing of mice with OG-98 and RAD001 was by oral gavage. Details of transplant procedures and unblinded experiments are in the Supplementary Information.

### Lentiviral KD, CRISPR screening and RNA sequencing

Lentiviral supernatants were prepared and cells were infected as previously described [4]. Details of specific vectors are in the Supplementary Information. Details of CRISPR screening, RNA sequencing and data analysis are in the Supplementary Information. RNA and sgRNA sequencing data are available at GEO with accession number GSE126486.

## Results

### Identification of genetic sensitizers to LSD1 inhibition in human THP1 AML cells

**Fig. 1:**
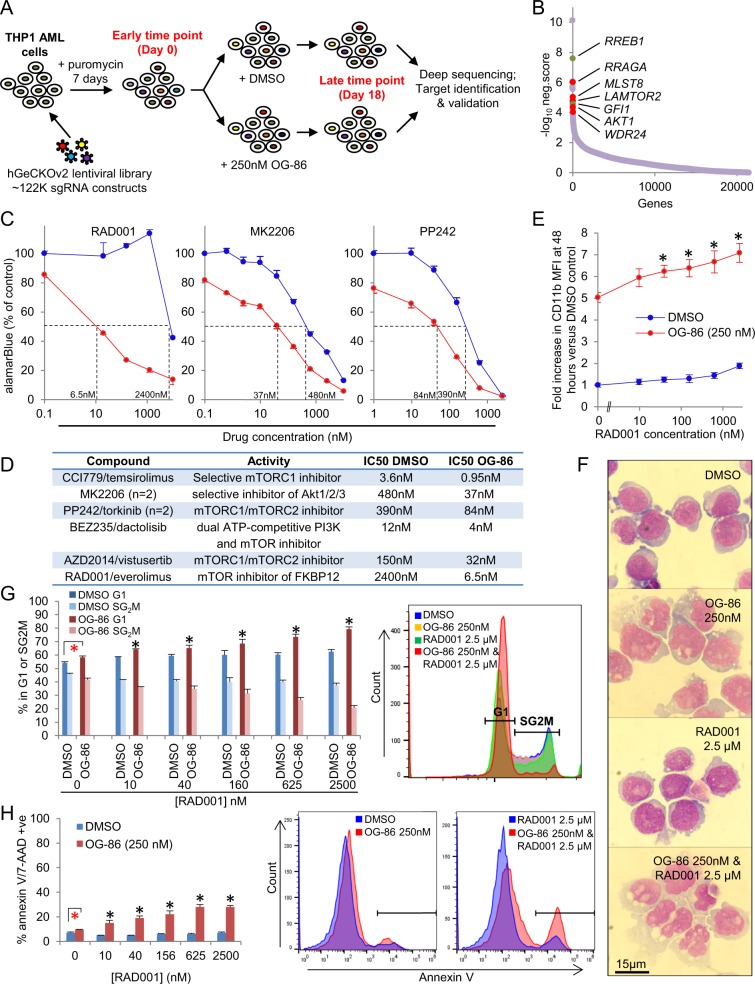
Identification of genetic sensitizers to LSD1 inhibition in human THP1 AML cells & combined pharmacologic inhibition of LSD1 and mTORC1. **a** Experimental outline. **b** Identification of top candidate genes using MAGeCK. **c** Relative alamarBlue signal from THP1 AML cells treated with OG-86 250 nM (red lines) or DMSO vehicle (blue lines) with MK2206, PP242 or RAD001 for 72 h (mean ± SEM; *n* = 3). **d** Summary of IC_50_ results. **e** CD11b expression with (**f**) indicative cytospin images of cellular morphology, (**g**) cell cycle analysis and (**h**) cellular viability (annexin V/7-AAD negative cells) in THP1 cells after 48 h (**e**, **g**) or 120 h (**f**, **h**) of treatment respectively with OG-86 250 nM (red lines) or DMSO vehicle (blue lines) and RAD001 (mean ± SEM; *n* = 3–6). For **e**, **g** and **h** black asterisks indicate *p* < 0.05 (one-way ANOVA, Fisher’s least significant difference post hoc test) for comparison of the indicated points with the OG-86 control condition. Red asterisks (**g**, **h**) indicate *p* < 0.05 (paired *t*-test) for the indicated comparison

To identify genes whose loss of function sensitizes cells to pharmacologic inhibition of LSD1, we performed a genome-wide loss-of-function CRISPR-Cas9 screen in human THP1 AML cells in the presence and absence of OG-86 (Oryzon Genomics, compound 86). OG-86 is a potent and specific tranylcypromine-derivative LSD1 inhibitor structurally related to and representative of inhibitors in clinical trials [1]. THP1 AML cells were selected because they exhibit a t(9;11) *MLL* gene rearrangement and respond to LSD1 inhibition in a similar manner to primary patient *MLL*-translocated AML cells, with differentiation and loss of clonogenic activity [8, 9]. Cells were infected with a pooled lentivirally expressed genome-scale CRISPR-Cas9 knockout (GeCKO) library, which targets 19,050 protein coding genes and 1864 microRNA precursor genes with 123,411 unique guide sequences (6 sgRNAs per gene) [11]. Prior to use, uniform library sgRNA representation and complexity was confirmed (Fig. S1A). Cells were infected in replicates with a low multiplicity of infection (~0.3) and representation (cells per lentiviral construct) was 500-fold. Infected cells were selected with puromycin and treated with 250 nM OG-86 or DMSO vehicle for 18 days (Fig. 1a). We obtained ~20 million mapped reads per sample indicating that an average of 100 cells was transduced with each sgRNA (Fig. S1B). To confirm screen quality, we compared sgRNA representation between day 0 technical replicates (Fig. S1C), and also between day 0 cells and the plasmid library (Fig. S1D); both were strongly correlated. For additional quality control, we searched for genes whose sgRNA representation was depleted in both day 18 samples vs. the day 0 sample. At a false discovery rate of 10% model-based analysis of genome-wide CRISPR/Cas9 knockout (MAGeCK [12]) identified 369 genes (Table S1). These included the critical myeloid lineage transcription factor genes *SPI1* and *CEBPA* and overall 61% were core essential genes (Fig. S1E) [13] demonstrating that the screening strategy robustly read out genes with important cellular functions.

We next searched for guides selectively depleted in OG-86-treated vs. vehicle-treated cells. At false discovery rates of 5 and 10%, we identified 22 and 44 expressed genes respectively whose sgRNA representation was depleted. These included genes with protein products predicted to regulate the structure or function of chromatin, act as transcription factors or act in metabolism (Table S2). With the exceptions of *CDC7* and *KCNQ4*, there was no overlap of the 44 genes with the 369 genes whose sgRNA representation was depleted in both day 18 samples vs. the day 0 sample, suggesting that LSD1 inhibition created novel genetic dependencies rather than exacerbating existing ones. Given our recent finding that inhibitors of LSD1 promote myeloid differentiation in *MLL*-translocated AML through disruption of the protein:protein interaction of the transcription repressor GFI1 with LSD1 [9], it was of note that guides targeting *GFI1* and the LSD1/CoREST complex gene *RREB1* scored highly in the screen (Fig. 1b). Combined targeting of the different components of the complex may prove more effective in promoting differentiation of AML cells than LSD1 inhibition alone.

Most significantly, guides targeting genes coding for multiple positive regulators of mTORC1 signalling were depleted, including *MLST8*, *RRAGA*, *LAMTOR2*, *WDR24* and *AKT1* (Fig. 1b). The mTORC1 complex controls the balance of anabolism vs. catabolism according to prevailing environmental conditions [14]. MLST8 is a core component of mTORC1, the GTPase RRAGA facilitates recruitment of mTORC1 to the surface of lysosomes following amino acid stimulation, RAG proteins are tethered to the lysosomal membrane by association with the pentameric Ragulator complex of which LAMTOR2 is a member, WDR24 is a component of the GATOR2 complex that activates mTORC1 in response to cytosolic arginine and the serine/threonine kinase AKT1 indirectly activates mTORC1 through phosphorylation of TSC2 and PRAS40 [14].

### Combined pharmacologic inhibition of LSD1 and mTORC1 impairs AML cell growth

To validate these observations, we targeted exemplar genes *RRAGA* and *MLST8* for KD in THP1 AML cells (Fig. S2A) and cultured control or KD cells in the presence or absence of OG-86 (Fig. S2B). Treatment of control cells with OG-86 impairs growth through rapid induction of a myeloid differentiation programme (marked by cell surface proteins CD11b and CD86), a decrease in the proportion of cycling cells and a slight increase in apoptosis [9] (Fig. S2B–H). Concomitant *RRAGA* or *MLST8* KD significantly reduced cell growth vs. control cells in the OG-86 condition, with the most notable difference being significant additional up regulation of CD11b (but not CD86) (Fig. S2D–F). *RRAGA* KD cells cultured in vehicle conditions exhibited reduced growth by comparison with control cells, a reduced proportion of cells in SG_2_M, increased CD11b expression and increased apoptosis. Upregulation of CD11b without changes in cell growth was also noted in control conditions for one of the *MLST8* KD constructs (Fig. S2G, H). Our observation of enhanced expression of CD11b in OG-86-cultured KD cells raised a question as to whether concomitant pharmacologic targeting of mTORC1 and LSD1 might collaborate to further induce a molecular differentiation programme in AML cells.

To address this, cells were treated with mTORC1 inhibitors, or the AKT inhibitor MK2206, in the presence or absence of 250 nM OG-86. OG-86 lowered the IC_50_ for growth in the presence of the various inhibitors 3–13 fold, and in the case of the highly selective mTORC1 inhibitor RAD001 by ~350-fold (Fig. 1c, d). There were dose dependent increases in expression of CD11b, the proportion of cells in G1 and in apoptosis in cells treated with RAD001 and 250 nM OG-86 (Fig. 1e–h). Similar findings for CD11b expression were observed in cells treated with MK2206 and PP242 (Fig. S3A, B). As expected, treatment of cells with RAD001 reduced phosphorylation of the mTORC1 downstream targets p70 S6 kinase and eukaryotic translation initiation factor 4E-binding protein 1 whether in the presence or absence of OG-86. However, in the presence of OG-86, and consistent with reduced cellular growth in the combination condition, the activity of phospho-ERK MAP kinase (but not AKT (data not shown)) signalling was reduced (Fig. S3C, D).

Given these genetic and pharmacologic findings, and the role of mTORC1 in amino acid sensing, we next evaluated whether culture of cells in media depleted of individual amino acids might alter expression of cellular markers associated with differentiation. Removal of essential amino acids from media, or non-essential amino acids glutamine, arginine or tyrosine alone substantially reduced growth and impaired cellular viability (Fig. S4A, B). Interestingly, removal of methionine or arginine, whose presence is sensed by a mechanism involving lysosomal RRAGA and mTORC1 [15], led to upregulation of the differentiation marker CD86 (but not CD11b) in both the absence and presence of OG-86 (Fig. S4C, D).

Altogether these data demonstrate that THP1 AML cells treated with LSD1 inhibition are sensitized to KD or pharmacologic inhibition of mTORC1 signalling.

### Synergistic activation of a myeloid differentiation programme by combined pharmacologic inhibition of LSD1 and mTORC1

**Fig. 2:**
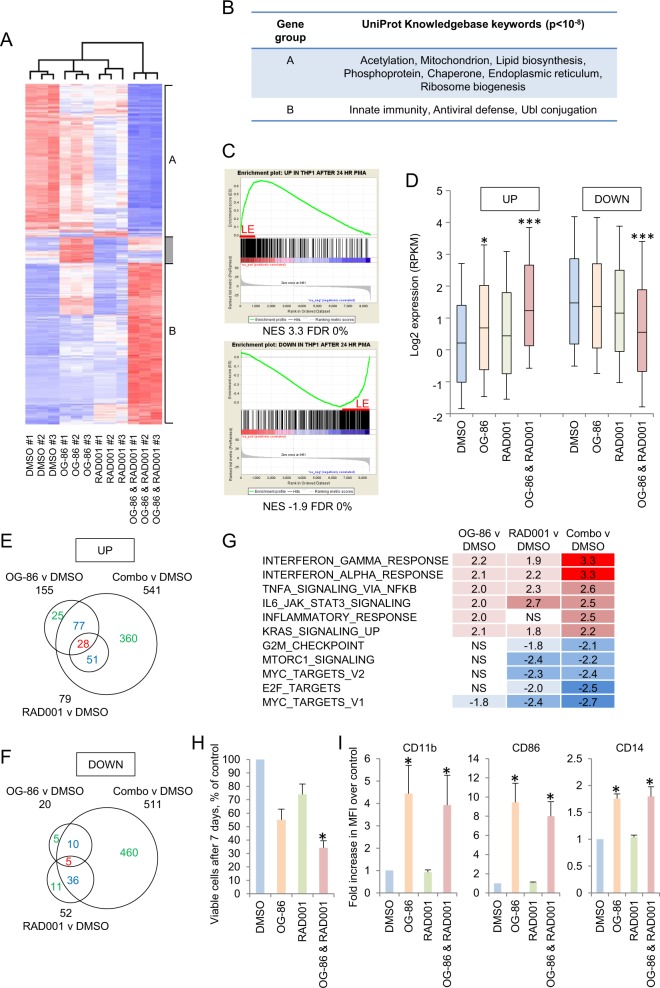
Synergistic activation of a myeloid differentiation programme in vitro by combined pharmacologic inhibition of LSD1 and mTORC1. **a**–**g** THP1 AML cells were treated for 24 h with DMSO vehicle, OG-86 (250 nM), RAD001 (40 nM) or a combination of both. **a** Heat map shows hierarchical cluster analysis of differentially expressed genes (i.e. *p* < 0.001 by one-way ANOVA, mean fold change >2 in at least one of the six pairwise comparisons). Downregulated (*n* = 551; Group A) and upregulated (*n* = 541; Group B) genes in the combination vs. the DMSO groups are indicated. **b** Keyword annotations of gene groups. **c** GSEA plots show enrichment of the indicated gene sets in OG-86/RAD001 combination vs. DMSO vehicle-treated transcriptomes. NES normalized enrichment score, FDR false discovery rate, LE leading edge. **d** Box plot shows median, 25th/75th (box) and 10th/90th (whiskers) centile distributions of leading edge gene expression values from **c**. *p* values for comparison of drug treated with DMSO vehicle-treated cells are indicated by *p < 0.05, ***p < 0.001 (one-way ANOVA with Fisher’s least significant difference post hoc test). Venn diagrams show overlap between **e** upregulated and **f** downregulated genes in the indicated comparisons. **g** Summary of GSEA analyses using the MSigDB hallmark gene set collection. NS not significant, FDR < 1.8. **h**, **i** Primary patient MLL-translocated AML cells were thawed and treated with DMSO vehicle or the indicated compounds in stromal co-culture for 7 days. **h** Relative cell numbers and **i** relative expression of cell surface differentiation markers, as determined by flow cytometry. An asterisk indicates *p* < 0.05 by one-way ANOVA with Fisher’s least significant difference post hoc test

To evaluate combined mTORC1 and LSD1 inhibition, we next performed RNA sequencing of THP1 AML cells treated for 24 h with DMSO vehicle, OG-86 (250 nM), RAD001 (40 nM) or a combination of both. One thousand one hundred fifty nine genes met criteria for significant differential expression across the four sample groups (i.e. *p* < 0.001 by one-way ANOVA, mean fold change in expression >2 in at least one of the six pairwise comparisons) (Table S3). Hierarchical cluster analysis (Fig. 2a) revealed clear separation of OG-86/RAD001-treated cells from the other groups. Clustering was driven by two groups of genes: Group A genes were downregulated in OG-86/RAD001-treated vs. vehicle-treated cells, while Group B genes were upregulated (Fig. 2a and Table S3). The Group A set was enriched for genes annotated with UniProt Knowledgebase keywords associated with active metabolism, whereas the Group B set was enriched for terms associated with immune function (Fig. 2b), further suggesting that the combination treatment much more potently induced a differentiation programme than the single treatments.

Gene set enrichment analysis (GSEA) using gene sets up- or downregulated during phorbol ester (PMA)-induced terminal differentiation of THP1 AML cells into macrophages (Table S4) [16, 17] demonstrated that the transcriptional changes observed in OG-86/RAD001-treated vs. vehicle-treated cells were similar to those up- and downregulated during differentiation. Leading edge analysis (Fig. 2c, d) showed that the expression of the upregulated leading edge gene set was significantly higher in the OG-86/RAD001 condition vs. all other conditions, and also in the OG-86 condition vs. DMSO vehicle-treated control cells (Fig. 2d). Expression of the downregulated leading edge gene set was significantly lower in the OG-86/RAD001 condition vs. all other conditions. Thus, while treatment of THP1 AML cells with OG-86 induces upregulation of a differentiation-associated gene set as previously reported [9], the combination treatment synergistically induced differentiation-associated transcriptional changes. This is exemplified by the observation that the set of genes significantly upregulated by OG-86 alone (including differentiation markers CD11b/ITGAM, CD86 and CD14; Table S3) was largely a subset of that upregulated by the OG-86/RAD001 combination treatment, and the set of genes upregulated by RAD001 alone entirely so (Fig. 2e). A similar pattern was noted among genes exhibiting significant downregulation (Fig. 2f).

We also explored transcriptional changes using GSEA and the Molecular Signatures Database hallmark gene set collection, each of which conveys a specific biological state or process and displays coherent expression [18]. Immune-associated gene sets characteristic of terminally differentiated monocytes and macrophages (e.g. NF-kB-induced TNF signalling and IFN-γ signalling, processes crucial for monocyte/macrophage activity during inflammation), were most strongly enriched in the OG-86/RAD001 vs. DMSO comparison, while those characteristic of cycling, metabolically active cells were most strongly depleted (Fig. 2g). Interestingly, we also observed the strongest depletion of a gene set associated with leukaemia stem cell maintenance in murine MLL-AF9 AML cells in the OG-86/RAD001 vs. DMSO comparison, as well as the strongest upregulation of genes whose expression is anti-correlated with leukaemia stem cell activity (Fig. S5A–C, Table S4) [19].

### In vivo analysis of combined pharmacologic inhibition of LSD1 and mTORC1

To evaluate whether these cell line observations were applicable in patient cells, we treated primary *MLL*-translocated AML cells from seven separate individuals (Table S5) for 7 days in stromal co-culture with vehicle, OG-86 250 nM, RAD001 2 μM or the combination. While RAD001 or OG-86 alone significantly reduced expansion of cell numbers relative to control cells, the combination reduced expansion of cell numbers significantly further by comparison with both individual conditions (Fig. 2h). By immunophenotyping, OG-86 induced significant upregulation of myeloid differentiation markers CD11b, CD86 and CD14, although there was no difference when the OG-86 alone and combination treatments were compared (Fig. 2i).

**Fig. 3:**
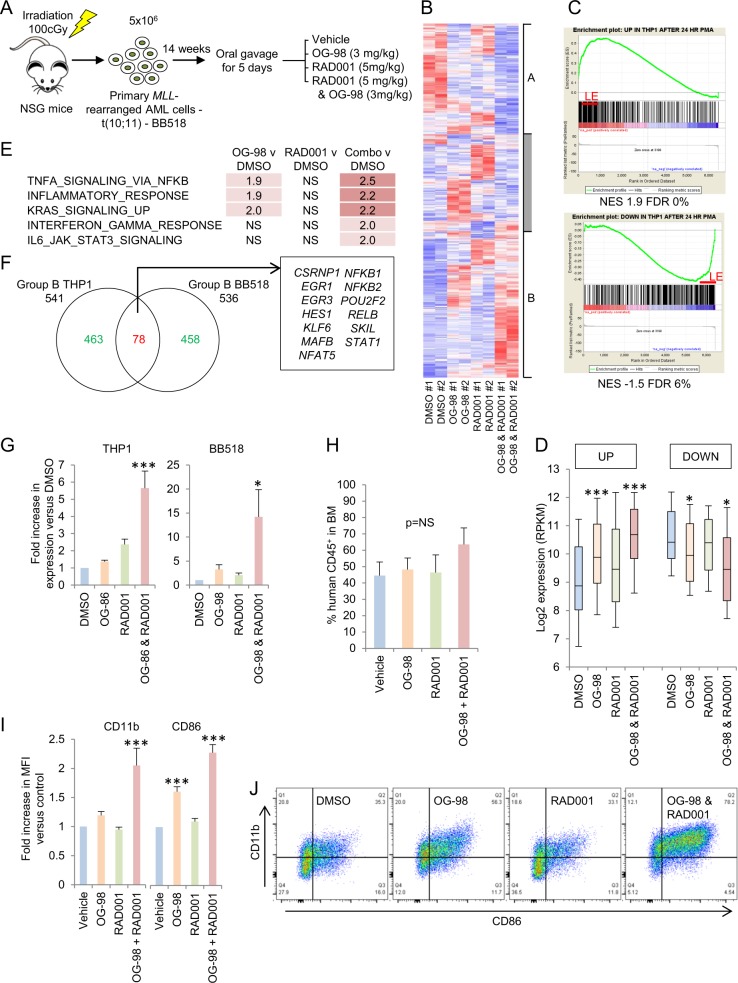
Synergistic activation of a myeloid differentiation programme in vivo by combined pharmacologic inhibition of LSD1 and mTORC1. **a** Experimental outline. **b**–**f** Bone marrow derived human CD45^+^ cells were immunomagnetically sorted and subjected to RNA sequencing. **b** Heat map shows hierarchical cluster analysis of differentially expressed genes (i.e. *p* < 0.05 by one-way ANOVA, mean fold change >1.5 in at least one of the six pairwise comparisons). Downregulated (*n* = 428; Group A) and upregulated (*n* = 536; Group B) genes in the combination vs. the vehicle groups are indicated. **c** GSEA plots show enrichment of the indicated gene sets in OG-98/RAD001 combination vs. vehicle-treated transcriptomes. NES normalized enrichment score, FDR false discovery rate, LE leading edge. **d** Box plot shows median, 25th/75th (box) and 10th/90th (whiskers) centile distributions of leading edge gene expression values from **c**. **e** Summary of GSEA analyses using the MSigDB hallmark gene set collection. NS not significant; FDR < 1.8. **f** Venn diagram shows overlap in Group B upregulated genes in THP1 cells and primary patient *MLL*-translocated cells, with transcription factor genes highlighted. **g** Mean ± SEM relative expression of the 13 transcription factors highlighted in **f** in THP1 cells in vitro and primary patient *MLL*-translocated cells in vivo. **h** Bar chart shows mean ± SEM percentage human CD45^+^ cells in bone marrow at euthanasia (*n* = 7 per cohort). NS not significant. **i** Bar chart shows mean ± SEM expression of CD11b and CD86 by human CD45^+^ cells, as determined by flow cytometry. **j** Representative flow cytometry plots. For **d**, **g** and **i** *p* values for comparison of drug treated vs. vehicle-treated cells are indicated by *p < 0.05 or ***p < 0.001 (one-way ANOVA with Fisher’s least significant difference post hoc test)

To determine whether our in vitro observations were also seen in vivo, we evaluated the effect of the combination treatment on xenoengrafted primary *MLL*-translocated AML cells exhibiting a substantial disease burden. NSG mice were sub-lethally irradiated and each injected with 5 × 10^6^ primary AML cells from a patient with a t(10;11) translocation (Fig. 3a). Fourteen weeks later, when circulating human AML cells could be detected in a majority of animals, mice were allocated to four groups balanced for human CD45^+^ blood chimerism and treated via oral gavage with either vehicle (H_2_O), RAD001 (5 mg/kg), OG-98 (3 mg/kg) or a combination of RAD001 (5 mg/kg) and OG-98 (3 mg/kg) for 5 days. OG-98 (2-((trans-2-(4-(benzyloxy)phenyl)cyclopropyl)amino)-1-(4-methylpiperazin-1-yl)ethanone) (Fig. S6A) is an orally bioavailable, irreversible, tranylcypromine-derivative inhibitor of LSD1 with high selectivity for LSD1 vs. the amine oxidases MAOA and MAOB, low-mid nanomolar potency (Fig. S6B) and a plasma half-life following oral dosing of 3.27 h (Fig. S6C). It is a cyclopropylamino-based LSD1 inhibitor similar to OG-86 (Fig. S6A) but with superior oral bioavailability making it suitable for in vivo analyses. At the end of treatment, human CD45^+^ cells were recovered from murine bone marrow by immunomagnetic selection and, following confirmation of population purity, RNA sequencing was performed.

As for THP1 cells, the greatest transcriptional changes were observed between AML cells recovered from mice treated with the drug combination vs. vehicle. 1368 genes met criteria for significant differential expression across the four sample groups (i.e. *p* < 0.05 by one-way ANOVA, mean fold change in expression >1.5 in at least one of the six pairwise comparisons) (Table S6). Hierarchical cluster analysis (Fig. 3b) revealed clear separation of OG-98/RAD001-treated cells from the other groups. Clustering was again driven by Group A genes, which were downregulated in OG-86/RAD001-treated, vs. vehicle-treated cells and Group B genes, which were upregulated (Fig. 3b and Table S6). GSEA using gene sets up- or downregulated during PMA-induced terminal differentiation of THP1 AML cells into macrophages (Table S4) [17] demonstrated that the transcriptional changes observed in OG-98/RAD001-treated vs. DMSO vehicle-treated primary *MLL*-translocated AML cells were similar (Fig. 3c). Leading edge analysis (Fig. 3c, d) revealed that expression of the upregulated leading edge gene set was significantly higher in OG-98/RAD001-treated cells vs. all other conditions, and also in OG-98 vs. vehicle-treated cells (Fig. 3d). Expression of the downregulated leading edge gene set was significantly lower in the OG-98/RAD001 condition vs. all other conditions, and also in the OG-98 condition vs. vehicle-treated control cells (Fig. 3d). As for THP1 cells (Fig. 2g), immune-associated gene sets characteristic of terminally differentiated monocytes and macrophages were most strongly upregulated in AML cells recovered from mice treated with the OG-98/RAD001 combination (Fig. 3e); similar findings were noted for genes whose expression is anti-correlated with MLL-AF9 leukaemic stem cell activity (Fig. S5D–F). Thus the transcriptional changes observed in *MLL*-translocated THP1 AML cells in vitro were similar to those observed in primary *MLL*-translocated patient cells in vivo. Specifically, there was significant overlap of the Group B gene sets upregulated following combination treatment in THP1 AML cells and primary patient AML cell xenografts by comparison with control cells (Fig. 3f), and these genes included those coding for 13 transcription factors including those with significant roles in monocyte/macrophage differentiation, such as *MAFB*, *KLF6*, *EGR1* and *EGR3*, although not *IRF8* [20]. Indeed in both THP1 and primary AML cells upregulation of expression of this set of 13 transcription factor genes was synergistic in the combination treatment vs. the individual treatments or control (Fig. 3g). In keeping with the transcriptional changes, while there was no significant difference in human CD45^+^ chimerism in bone marrow in the four groups (the cohorts were balanced ahead of drug treatment for blood CD45^+^ chimerism), there was a significant increase in cell surface expression of the differentiation markers CD11b and CD86 in the combination treated vs. all other cohorts, and in expression of CD86 in OG-98 vs. vehicle-treated mice (Fig. 3h–j). Thus in in vitro and in vivo analyses of *MLL*-translocated cell line and primary samples, concomitant pharmacologic inhibition of LSD1 and mTORC1 induces synergistic upregulation of a monocyte/macrophage differentiation programme.

**Fig. 4:**
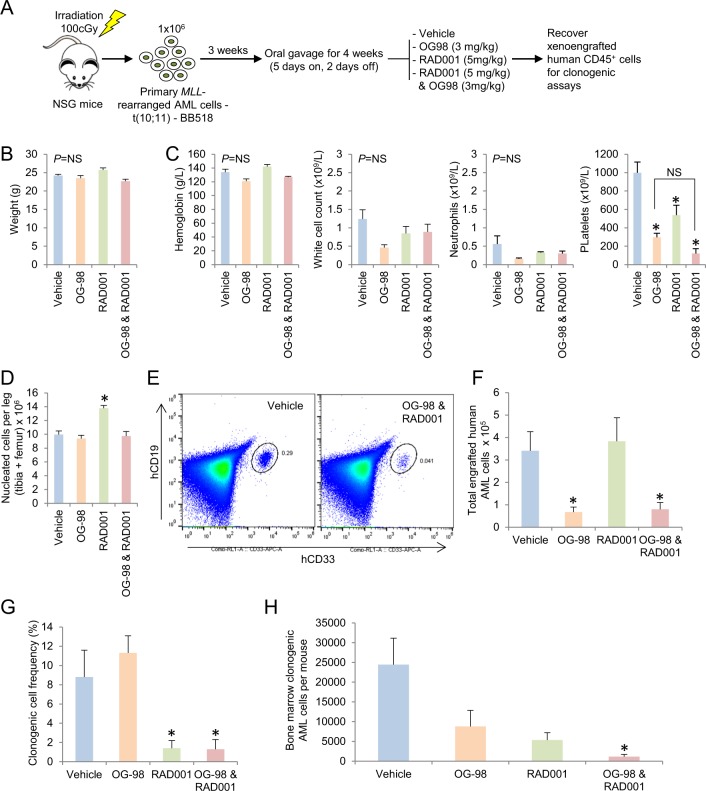
Combined pharmacologic inhibition of LSD1 and mTORC1 impairs primary AML cell clonogenic activity in vivo. **a** Experimental outline. **b** Mouse weights, **c** blood counts and **d** bone marrow nucleated cells in one leg at end of treatment (mean ± SEM; *n* = 5 per cohort). Statistical significance was assessed by one-way ANOVA with Fisher’s least significant difference post hoc test. An asterisk indicates *p* < 0.05 for comparison of indicated cohort with DMSO control cohort. **e** Example flow cytometry plots. **f** Total engrafted human AML cells, **g** the clonogenic cell frequency of those populations and **h** total bone marrow clonogenic AML cells in the indicated cohorts (mean ± SEM; *n* = 5 per cohort). An asterisk indicates *p* < 0.05 (one-way ANOVA with Fisher’s least significant difference post hoc test) (**f**, **g**) for comparison of indicated cohort with DMSO control cohort or (**h**) for comparison of OG-98/RAD001 cohort with all other cohorts

A potential clinical scenario for use of combined LSD1 and mTORC1 inhibition is to promote differentiation of residual clonogenic AML cells in a minimal residual disease setting after, for example, completion of intensive chemotherapy. To evaluate this possibility we transplanted NSG mice with a low burden of primary AML cells (10^6^ per mouse) and 3 weeks later treated animals with drugs for 4 weeks (Fig. 4a; *n* = 5 per cohort). The RAD001/OG-98 combination was well tolerated: at the end of treatment there was no significant difference in weight, haemoglobin, total white cell count or neutrophil count (Fig. 4b, c). There was, as expected [7], a significant reduction in platelet count in all cohorts compared with controls, although the difference between the RAD001/OG-98 combination and OG-98 only cohorts was not statistically significant (Fig. 4c). Bone marrow cellularity was significantly higher in the RAD001-treated cohort for unclear reasons (Fig. 4d). Across all cohorts the median percentage of human CD33^+^ AML cells engrafted in murine bone marrow was 0.13 (range 0.029–0.51) (Fig. 4e). Based on the frequency of human AML cells in each mouse, the total bone marrow cell count from one tibia and femur (Fig. 4d) and the estimation that bone marrow from one leg accounts for 10% of total murine marrow [21], we calculated the total number of bone marrow engrafted primary human AML cells per mouse (Fig. 4f). To evaluate the functional potential of engrafted human AML cells, we recovered them using anti-human CD45 immunomagnetic selection and performed clonogenic assays (Figs. 4g, S7A). Flow analysis of cells recovered from clonogenic assays confirmed human myeloid origin of nearly all cells (Fig. S7B–D). The clonogenic cell frequencies enabled calculation of the total number of clonogenic bone marrow engrafted primary human AML cells per mouse. We found that OG-98 reduced the number of engrafted AML cells (Fig. 4f), and that RAD001 reduced the clonogenic activity of those engrafted cells (Fig. 4g). The consequence of this was that the OG-98/RAD001 combination-treated cohort exhibited significantly fewer bone marrow engrafted clonogenic AML cells by comparison with all other cohorts (a mean of 21.1-fold, 7.6-fold and 4.6-fold fewer than in the DMSO, OG-98 or RAD001-treated cohorts, respectively) (Fig. 4h).

Finally, given the activity of combinatorial LSD1 and mTORC1 inhibition in *MLL*-leukaemias we evaluated OG-86 and RAD001 in primary AML cells of additional genotypes in stromal co-culture (Fig. S8A, B). Interestingly, while OG-86 alone had little overall impact on cell growth, RAD001 showed moderate anti-proliferative activity, and the combination once more significantly impaired cell growth by comparison with the other conditions.

## Discussion

The choice of which therapeutic combinations to test in clinical trials is often empiric. Genetic screening approaches such as CRISPR hold out promise for more rational decisions. Using a CRISPR-based screening approach with confirmatory KD, pharmacologic and amino acid deprivation experiments we find that *MLL*-translocated AML cells exhibit increased sensitivity to mTORC1 inhibition in the presence of pharmacologic inhibition of LSD1. Importantly, the combination leads to synergistic upregulation of a molecular differentiation programme, which includes key transcription factors involved in monocytic lineage differentiation. The cardinal feature of AML is a block to normal differentiation and treatments which release that block, such as all-trans retinoic acid (ATRA) in acute promyelocytic leukaemia, are essential components of therapeutic regimens. Induction of differentiation is often coupled with loss of self-renewal in AML; novel combinations that promote differentiation may therefore also target leukaemic stem cells.

Prior studies have reported additive or synergistic anti-leukaemic activity of drugs in combination with LSD1 inhibition. For example, ATRA in combination with tranylcypromine or related derivatives confers enhanced inhibition of growth, upregulation of differentiation markers or leukaemia cell engraftment in AML cell models [5, 22, 23], as does the NEDD8-activating enzyme inhibitor pevonedistat [24]. ORY-1001 exhibits various in vitro synergies in certain AML cell lines, including with ATRA, cytarabine, quizartinib, decitabine, azacitidine, vorinostat, the DOT1L inhibitor EPZ5676 and the BCL2 inhibitor ABT-737 [7]. SP2509, a reversible inhibitor of LSD1, is reported to exhibit synergy with either the HDAC inhibitor panobinostat or the EZH2 inhibitor EPZ6438 [25, 26], although whether the activity of SP2509 is on target remains unclear [27]. It is of note that in our screen we did not observe depletion of guides targeting genes coding for proteins involved in retinoic acid signalling, neddylation or, with the exception of *JARID2*, core polycomb repressive complex 2 components. By contrast, our genetic screen revealed a strong depletion signal for mTORC1 pathway genes.

mTOR signalling is elevated in AML and consequences include inhibition of autophagy and altered cell growth [28]. Clinical trials of rapamycin or rapalogs such as everolimus, alone or in combination with conventional chemotherapy, have demonstrated limited efficacy, even though well tolerated [28]. Our studies suggest that evaluation of combined LSD1 and mTORC1 inhibition in *MLL*-translocated AML, perhaps following intensive chemotherapy and remission induction, with the goal of inducing differentiation of minimal residual disease may prove beneficial.

LSD1 inhibition in *MLL*-translocated AML cells reduces activity of GFI1 through physical disruption of the LSD1/CoREST complex with GFI1 on chromatin [9]. GFI1 is a transcription repressor that counteracts the activity of the monocytic lineage transcription factor IRF8 [29]. The altered balance of these factors favours monocytic lineage differentiation. We found that the extent of leukaemia cell differentiation induced by LSD1 inhibitors was greatly enhanced by concomitant treatment of cells with rapamycin, although the mechanism underlying the synergistic upregulation of monocytic lineage transcription factor genes remains unclear. The mTOR pathway has been shown to be crucial for regulating the function of innate immune cell populations including monocytes, macrophages and dendritic cells [30]. However, mTORC1 seems to have divergent roles during activation and differentiation of myeloid cells dependent on the cell type. While activation of PI-3-kinase or mTORC1 seems to be important for survival and expansion of monocyte-derived dendritic cells, inhibition of mTOR signalling with rapamycin in myeloid progenitors favours the translation of more abundant inflammatory cytokines by enhanced activation of NF-kB, and expression of costimulatory molecules at the cell surface such as CD86, programmed death ligand-1 and CD25 [30]. It is interesting to note that following deletion in haematopoietic stem cells of *Rptor*, which codes for the substrate-guiding subunit of the mTORC1 complex, mice develop a modest pancytopenia with increased monocytoid cells in bone marrow and spleen [31] suggesting that mTORC1 signalling influences cell fate in granulocyte-monocyte/macrophage progenitor cells. Understanding the mechanism underlying the *Rptor* knockout phenotype will be key to understanding the synergy between LSD1 and mTORC1 inhibition in *MLL*-translocated and other AML cells. Of note, we did observe reduced phospho-ERK but not phospho-MEK signalling in AML cells treated with the combination raising a question as to whether the synergy arises at the level of the lysosome where scaffolding of MEK and ERK occurs and where too mTORC1 is located. Additional investigations will be revealing.

## Supplementary information

Supplemental information

Supplementary Tables

## References

[1] Maiques-Diaz A, Somervaille TC (2016). LSD1: biologic roles and therapeutic targeting. Epigenomics.

[2] Baron R, Binda C, Tortorici M, McCammon JA, Mattevi A (2011). Molecular mimicry and ligand recognition in binding and catalysis by the histone demethylase LSD1-CoREST complex. Structure.

[3] Saleque S, Kim J, Rooke HM, Orkin SH (2007). Epigenetic regulation of hematopoietic differentiation by Gfi-1 and Gfi-1b is mediated by the cofactors CoREST and LSD1. Mol Cell.

[4] Harris WJ, Huang X, Lynch JT, Spencer GJ, Hitchin JR, Li Y (2012). The histone demethylase KDM1A sustains the oncogenic potential of MLL-AF9 leukemia stem cells. Cancer Cell.

[5] Schenk T, Chen WC, Gollner S, Howell L, Jin L, Hebestreit K (2012). Inhibition of the LSD1 (KDM1A) demethylase reactivates the all-trans-retinoic acid differentiation pathway in acute myeloid leukemia. Nat Med.

[6] Mohammad HP, Smitheman KN, Kamat CD, Soong D, Federowicz KE, Van Aller GS (2015). A DNA Hypomethylation signature predicts antitumor activity of LSD1 inhibitors in SCLC. Cancer Cell.

[7] Maes T, Mascaro C, Tirapu I, Estiarte A, Ciceri F, Lunardi S (2018). ORY-1001, a potent and selective covalent KDM1A inhibitor, for the treatment of acute leukemia. Cancer Cell.

[8] Somervaille T, Salamero O, Montesinos P, Willekens C, Perez Simon JA, Pigneux A (2016). Safety, phamacokinetics (pk), pharmacodynamics (pd) and preliminary activity in acute leukemia of ORY-1001, a first-in-class inhibitor of lysine-specific histone demethylase 1A (LSD1/KDM1A): initial results from a first-in-human phase 1 study. Blood.

[9] Maiques-Diaz A, Spencer GJ, Lynch JT, Ciceri F, Williams EL, Amaral FMR (2018). Enhancer activation by pharmacologic displacement of LSD1 from GFI1 induces differentiation in acute myeloid leukemia. Cell Rep.

[10] Sanjana NE, Shalem O, Zhang F (2014). Improved vectors and genome-wide libraries for CRISPR screening. Nat Methods.

[11] Shalem O, Sanjana NE, Hartenian E, Shi X, Scott DA, Mikkelson T (2014). Genome-scale CRISPR-Cas9 knockout screening in human cells. Science.

[12] Li W, Xu H, Xiao T, Cong L, Love MI, Zhang F (2014). MAGeCK enables robust identification of essential genes from genome-scale CRISPR/Cas9 knockout screens. Genome Biol.

[13] Hart T, Chandrashekhar M, Aregger M, Steinhart Z, Brown KR, MacLeod G (2015). High-resolution CRISPR screens reveal fitness genes and genotype-specific cancer liabilities. Cell.

[14] Saxton RA, Sabatini DM (2017). mTOR signaling in growth, metabolism, and disease. Cell.

[15] Gu X, Orozco JM, Saxton RA, Condon KJ, Liu GY, Krawczyk PA (2017). SAMTOR is an S-adenosylmethionine sensor for the mTORC1 pathway. Science.

[16] Subramanian A, Tamayo P, Mootha VK, Mukherjee S, Ebert BL, Gillette MA (2005). Gene set enrichment analysis: a knowledge-based approach for interpreting genome-wide expression profiles. Proc Natl Acad Sci USA.

[17] Suzuki H, Forrest AR, van Nimwegen E, Daub CO, Balwierz PJ, Irvine KM (2009). The transcriptional network that controls growth arrest and differentiation in a human myeloid leukemia cell line. Nat Genet.

[18] Liberzon A, Subramanian A, Pinchback R, Thorvaldsdóttir H, Tamayo P, Mesirov JP (2011). Molecular signatures database (MSigDB) 3.0. Bioinformatics.

[19] Somervaille TC, Matheny CJ, Spencer GJ, Iwasaki M, Rinn JL, Witten DM (2009). Hierarchical maintenance of MLL myeloid leukemia stem cells employs a transcriptional program shared with embryonic rather than adult stem cells. Cell Stem Cell.

[20] Friedman AD (2007). Transcriptional control of granulocyte and monocyte development. Oncogene.

[21] Boggs DR (1984). The total marrow mass of the mouse: a simplified method of measurement. Am J Hematol.

[22] Binda C, Valente S, Romanenghi M, Pilotto S, Cirilli R, Karytinos A (2010). Biochemical, structural, and biological evaluation of tranylcypromine derivatives as inhibitors of histone demethylases LSD1 and LSD2. J Am Chem Soc.

[23] Smitheman KN, Severson TM, Rajapurkar SR, McCabe MT, Karpinich N, Foley J, et al. Lysine specific demethylase 1 inactivation enhances differentiation and promotes cytotoxic response when combined with all-trans retinoic acid in acute myeloid leukemia across subtypes. Haematologica. 2018. http://haematologica.org/content/early/2018/11/23/haematol.2018.199190. 10.3324/haematol.2018.199190 PMC6545850 30514804

[24] Ishikawa Y, Nakayama K, Morimoto M, Mizutani A, Nakayama A, Toyoshima K (2017). Synergistic anti-AML effects of the LSD1 inhibitor T-3775440 and the NEDD8-activating enzyme inhibitor pevonedistat via transdifferentiation and DNA rereplication. Oncogenesis.

[25] Fiskus W, Sharma S, Shah B, Portier BP, Devaraj SG, Liu K (2014). Highly effective combination of LSD1 (KDM1A) antagonist and pan-histone deacetylase inhibitor against human AML cells. Leukemia.

[26] Wen S, Wang J, Liu P, Li Y, Lu W, Hu Y (2018). Novel combination of histone methylation modulators with therapeutic synergy against acute myeloid leukemia in vitro and in vivo. Cancer Lett.

[27] Sonnemann J, Zimmermann M, Marx C, Ebert F, Becker S, Lauterjung ML (2018). LSD1 (KDM1A)-independent effects of the LSD1 inhibitor SP2509 in cancer cells. Br J Haematol.

[28] Herschbein L, Liesveld JL (2018). Dueling for dual inhibition: means to enhance effectiveness of PI3K/Akt/mTOR inhibitors in AML. Blood Rev.

[29] Olsson A, Venkatasubramanian M, Chaudhri VK, Aronow BJ, Salomonis N, Singh H (2016). Single-cell analysis of mixed-lineage states leading to a binary cell fate choice. Nature.

[30] Weichhart T, Hengstschlager M, Linke M (2015). Regulation of innate immune cell function by mTOR. Nat Rev Immunol.

[31] Kalaitzidis D, Sykes SM, Wang Z, Punt N, Tang Y, Ragu C (2012). mTOR complex 1 plays critical roles in hematopoiesis and Pten-loss-evoked leukemogenesis. Cell Stem Cell.

