# Supplementary material for: Pre-clinical activity of combined LSD1 and mTORC1 inhibition in MLL-translocated acute myeloid leukaemia
Source: Leukemia. 2019 Nov 28;34(5):1266–77. doi: 10.1038/s41375-019-0659-6 (PMC7192845; doi:10.1038/s41375-019-0659-6)
Supplement: Supplementary file 1 — Supplemental information [file 41375_2019_659_MOESM1_ESM.pdf]

Supplemental Information

Pre-clinical activity of combined LSD1 and mTORC1 inhibition in *MLL*-translocated acute myeloid leukaemia

Gauri Deb, Bettina Wingelhofer, Fabio M R Amaral, Alba Maiques-Diaz, John A Chadwick, Gary J Spencer, Emma L Williams, Hui-Sun Leong, Tamara Maes and Tim C P Somervaille

Table S5. Karyotypes of primary AML samples.

Biobank number	BM or PB	Karyotype
104	BM	46,XX,t(6;9;11)(p2?1;p22;q23)[6]/ 45,idem,der(15)t(15;17)(p11.2;q11.2),-17[4]
108	BM	46,XX,t(6;11)(q27;q23)[10]
148	PB	46,XY,t(6;11)(q27;q23)[10]/ 48,idem,+der(6)t(6;11),+21[4]
419	PB	46,XX,t(1;22)(p21;p11.2),ins(10;11)(p12;q23q1?4)[10] (MLL gene rearrangement confirmed by FISH)
514	BM	46,XX,t(9;11)(p22;q23)[1]/ 46,XX[7]
518	BM	45,XY,der(10)t(10;11)(p1?2;q23),der(10;12)(q10;q10),der(11)t(10;11)inv(11)(q21q23)[7]/ 45,XY,?add(7)(q?22),?add(10)(p?11.2),der(10;12)(q10;q10),der(11)t(10;11)inv(11)(q21q23),?i(17)(q10)[3]
582	BM	46,XX,der(1)t(1;1)(p36;q25),t(5;9;11)(q3?3;p22;q23)[5]/ 46,XX,t(5;9;11),add(17)(p11.2)[3]/46,XX,t(5;9;11),add(12)(q24)[2]

A

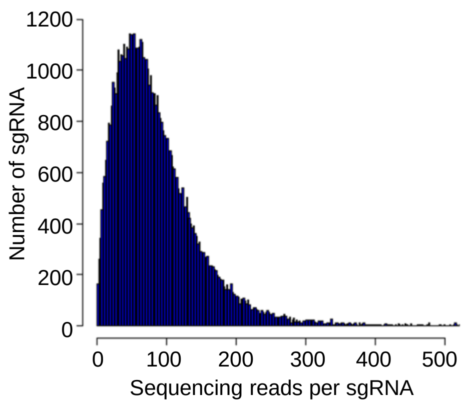

B

Sample	Total reads	Mapped reads	Filtered reads	sgRNAs with <10 reads	Coverage
Day 0 replicate1	10,579,497	9,067,220	8,616,700	7,127	73x
Day 0 replicate2	11,265,533	9,593,312	9,115,707	6,544	78x
D18 DMSO replicate 1	11,288,002	9,697,524	9,268,103	7,636	79x
D18 DMSO replicate 2	6,924,523	6,035,734	5,766,562	13,877	49x
D18 OG-86 replicate 1	9,634,375	8,345,528	7,975,895	9,745	68x
D18 OG-86 replicate 2	11,522,164	9,870,314	9,433,172	7,645	80x

C

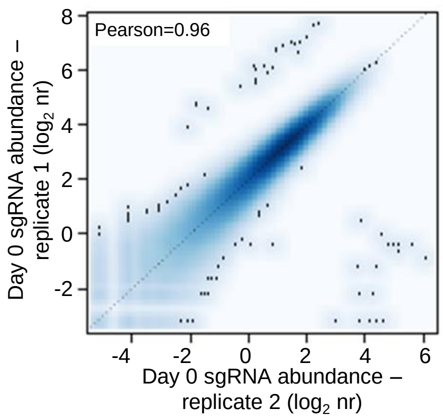

D

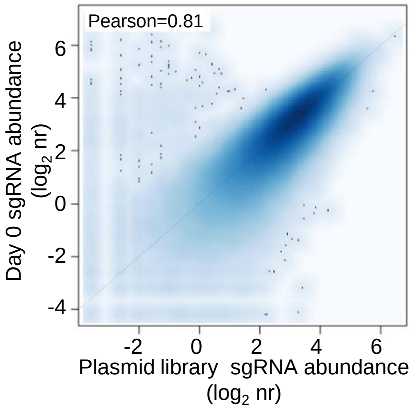

E

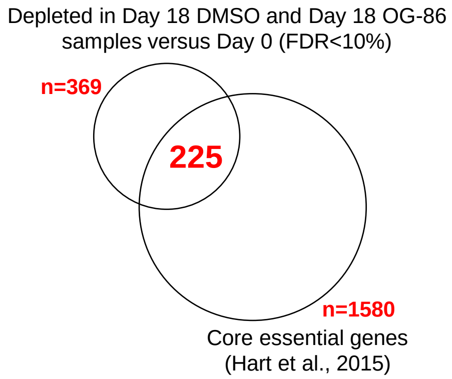

Figure S1. CRISPR screen quality control.

(A) Histogram shows distribution of sgRNA sequencing reads in the amplified GeCKOv2 library; 99% of sgRNAs were represented by at least one sequencing read. (B) Read statistics. Scatter plots show sgRNA representation between technical replicates of (C) early time point (Day 0) samples or (D) Day 0 versus plasmid pool samples (nr = normalized reads). (E) Venn diagram shows the overlap between genes depleted in both DMSO and OG-86 Day 18 samples by comparison with Day 0 and a reference 'core essential' gene set.[15]

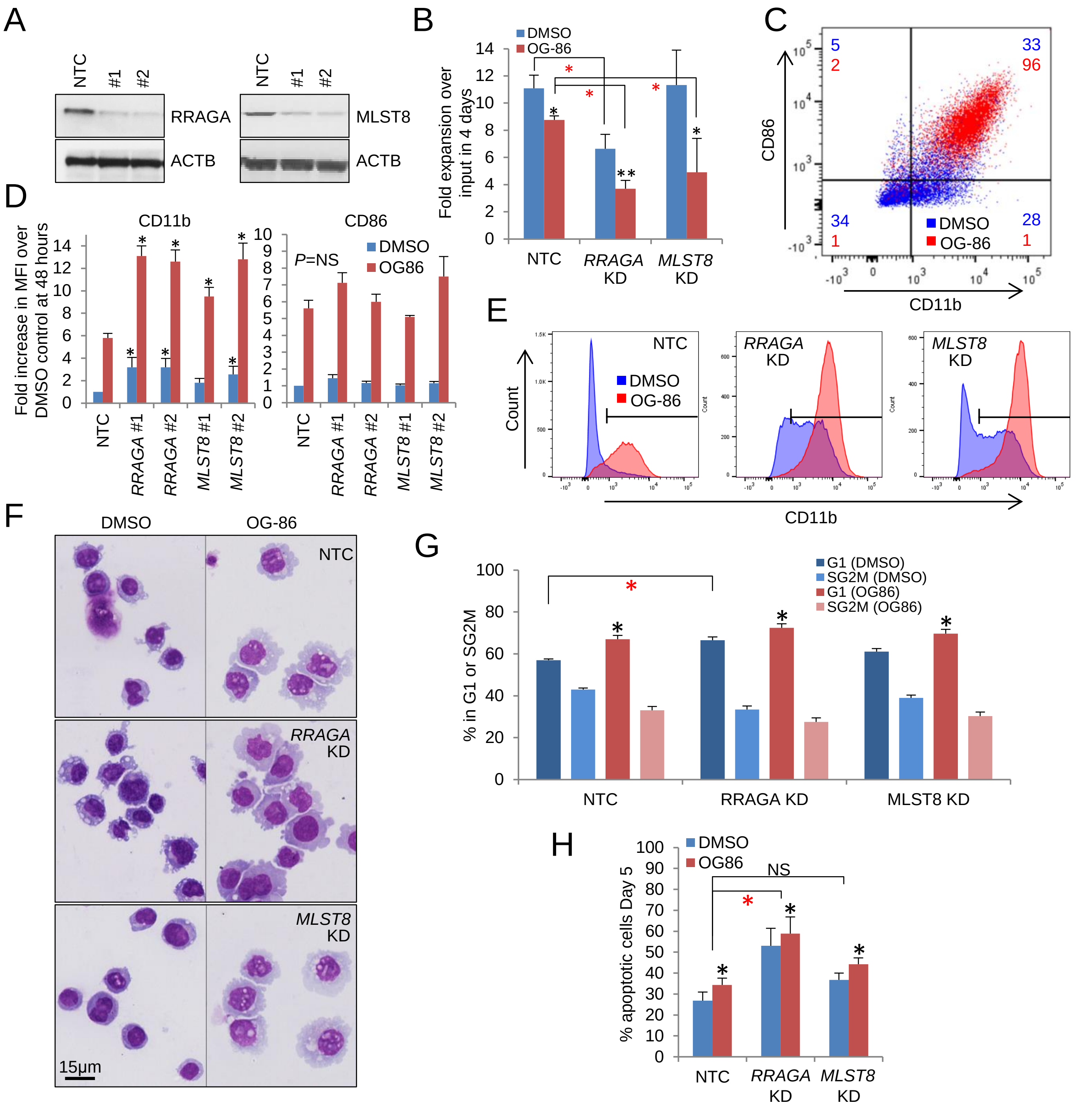

Figure S2. *RRAGA* and *MLST8* validation experiments in THP1 AML cells.

(A) Western blots show RRAGA and MLST8 knockdown 96hr following lentiviral infection. (B) Mean±SEM fold expansion over 96hr in liquid culture in the indicated conditions (n=4). *P*<0.05 or *P*<0.001 (t-test) indicated by * or ** respectively for OG-86 versus control condition. * indicates *P*<0.05 for the indicated comparisons (one way ANOVA with Fisher's least significant difference *post hoc* test). (C) Exemplar flow cytometry plot shows expression of the indicated markers after 48hr of drug treatment. (D) Mean±SEM (n=6) expression of CD11b and CD86 in the indicated conditions after 48hrs drug treatment with (E) exemplar flow cytometry plots. * indicates *P*<0.05 for comparison of the indicated vehicle or OG-86-treated condition versus their respective controls (one way ANOVA with Fisher's least significant difference *post hoc* test) (F) Exemplar cytospin preparations after seven days in liquid culture. Scale bar applies to all images. (G) Cell cycle and (H) apoptosis analysis of cells five days after initiation of knockdown and culture in the indicated conditions (mean±SEM; n=4); * indicates *P*<0.05 (paired t-test) for comparison of OG-86 condition with DMSO condition. * indicates *P*<0.05 (one way ANOVA with Fisher's least significant difference *post hoc* test) for comparison of RRAGA KD with NTC conditions. NS – not significant. NTC – non-targeting control. MFI – mean fluorescence intensity.

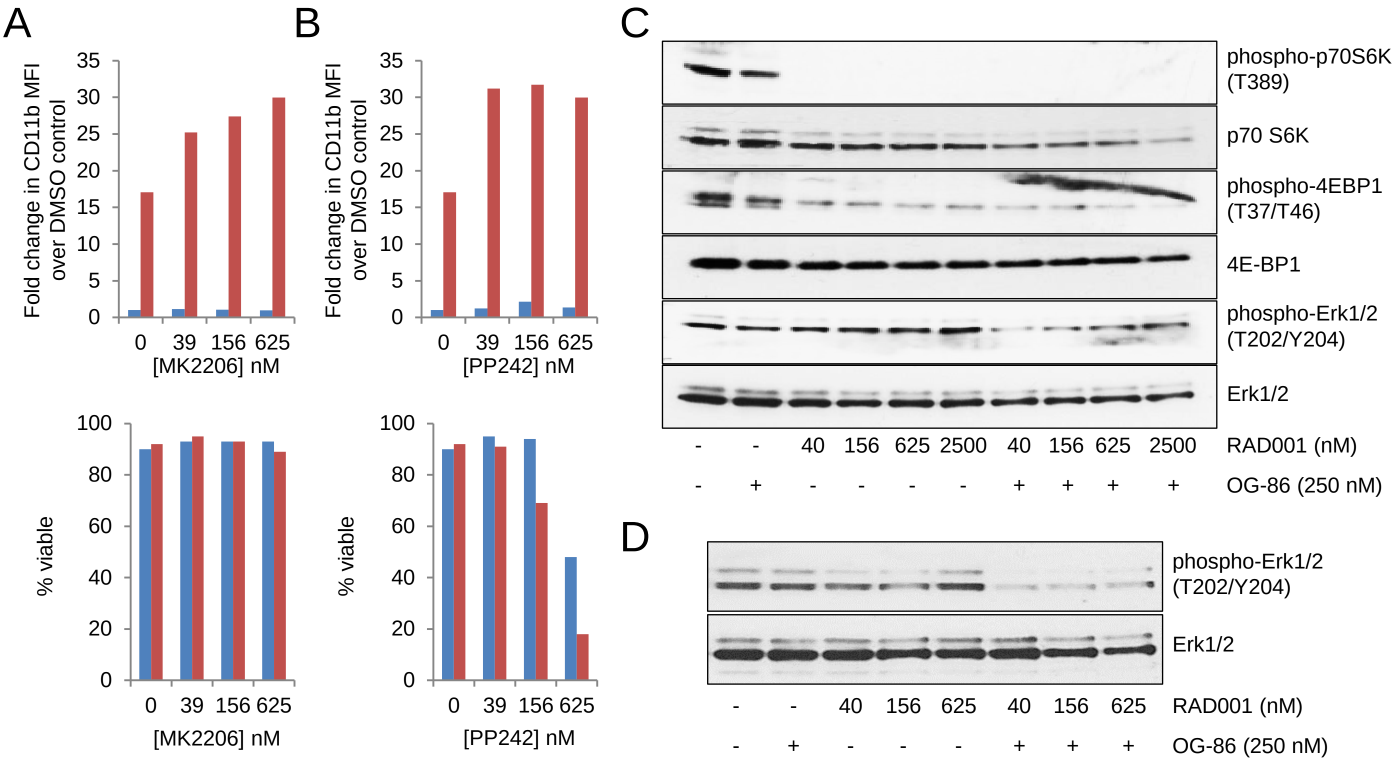

Figure S3. Combined pharmacologic inhibition of LSD1 and mTORC1 impairs THP1 AML cell growth.

CD11b expression (upper panels) and cellular viability (annexin V/7-AAD negative cells) (lower panels) in THP1 cells after 48hrs or 120hrs treatment respectively with OG-86 250nM (red bars) or DMSO vehicle (blue bars) and (A) MK2206 or (B) PP242. (C) & (D) Western blots for the indicated proteins under the indicated conditions.

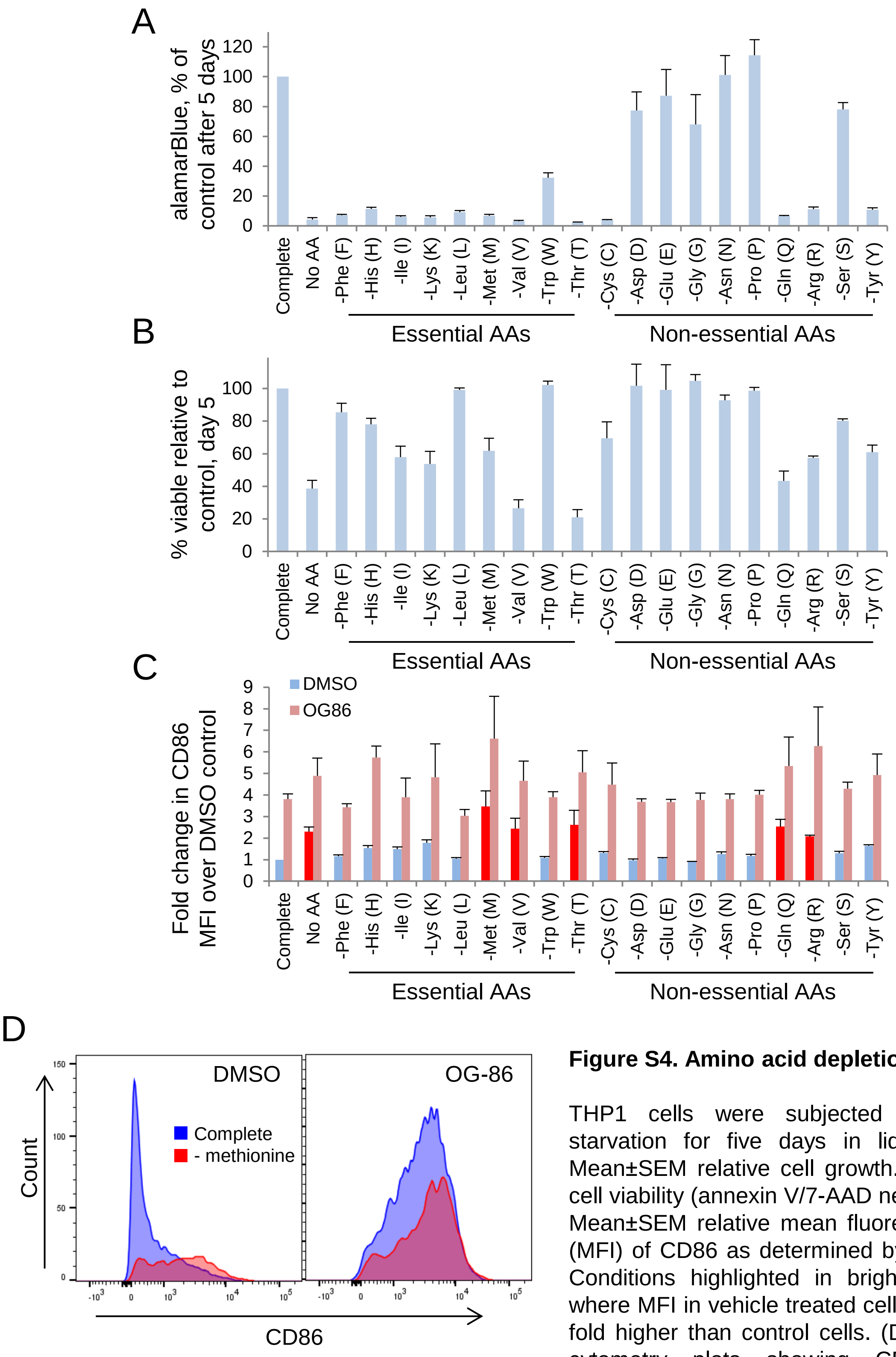

Figure S4. Amino acid depletion.

THP1 cells were subjected to amino acid starvation for five days in liquid culture. (A) Mean±SEM relative cell growth. (B) Mean±SEM cell viability (annexin V/7-AAD negative cells). (C) Mean±SEM relative mean fluorescence intensity (MFI) of CD86 as determined by flow cytometry. Conditions highlighted in bright red are those where MFI in vehicle treated cells is more than 2-fold higher than control cells. (D) Exemplar flow cytometry plots showing CD86 expression following methionine starvation for 48hr in the presence or absence of OG-86 250nM.

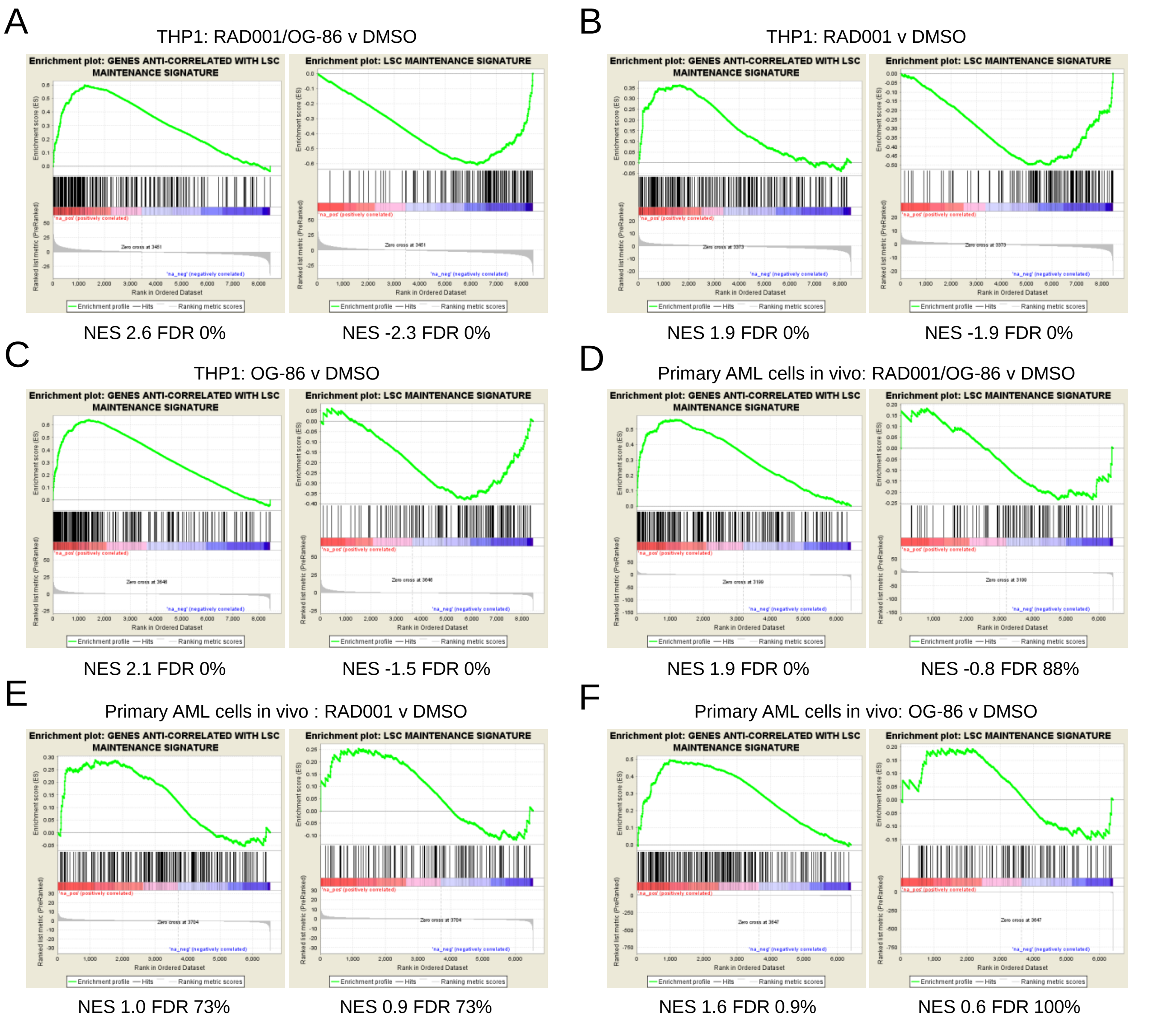

Figure S5. Gene set enrichment analyses.

Enrichment of gene sets positively and negatively correlated with leukemia stem cell potential in murine *MLL* leukemias (Table S4).

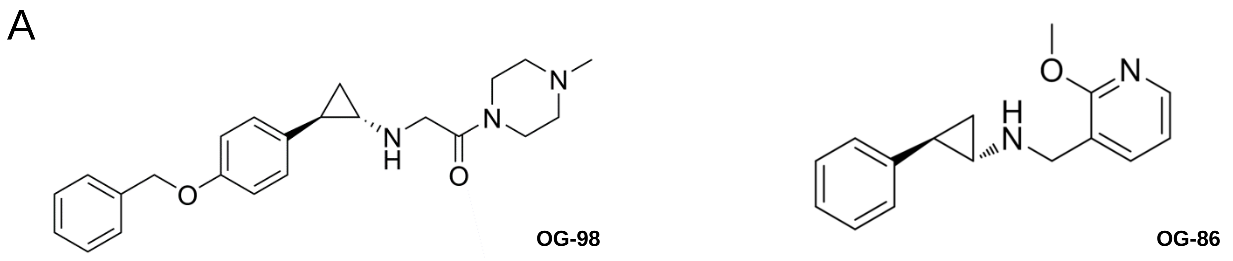

B

Enzyme	K_i	k_{inact}/K_i
LSD1	30nM	18750 $M^{-1}s^{-1}$
MAOA	11µM	244 $M^{-1}s^{-1}$
MAOB	9µM	4933 $M^{-1}s^{-1}$

C

	Value
Plasma T_{max}	60 min
Plasma C_{max}	14mg/mL
Plasma $t_{1/2}$	3.27 hr
AUC_{inf}	56.5 hr*ng/mL
Bioavailability	19%

Figure S6. Structure and properties of tranylcypromine-derivative inhibitors of LSD1.

(A) Structure of OG-98 (2-((trans-2-(4-(benzyloxy)phenyl)cyclopropyl)amino)-1-(4-methylpiperazin-1-yl)ethanone) and OG-86 (trans-N-((2-methoxypyridin-3-yl)methyl)-2-phenylcyclopropan-1-amine). (B) IC50 and K_{inact}/K_i values for OG-98 versus LSD1, MAOA and MAOB. (C) Pharmacokinetic data for OG-98 following a single oral 3 mg/kg dose delivered to CD1 mice (T_{max} - time to maximum plasma concentration; C_{max} - maximum concentration; $t_{1/2}$ - plasma half-life; AUC_{inf} - area under the curve extrapolated to infinity).

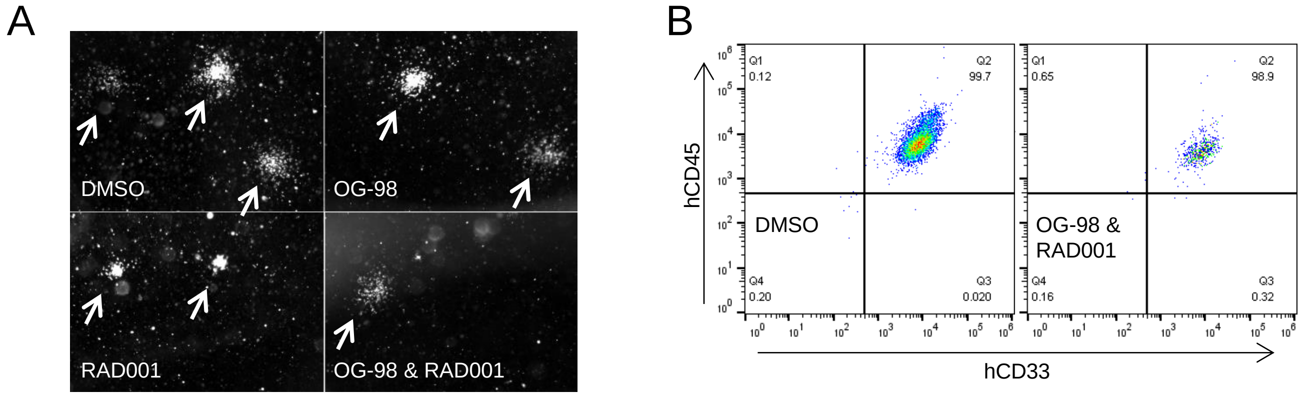

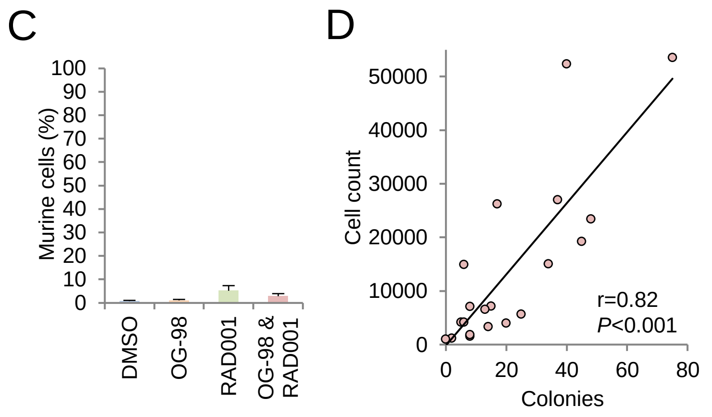

Figure S7. Minimal residual disease xenografts.

(A) Example images of primary human MLL leukaemia colonies. At the end of the colony assay, methylcellulose was washed out and cells counted and analysed for cell surface marker expression. (B) Example flow cytometry plots (C) Mean±SEM murine cells in semi-solid culture. (D) Scatter plot shows number of cells recovered from clonogenic assays correlates strongly with the number of colonies formed (n=20).

A

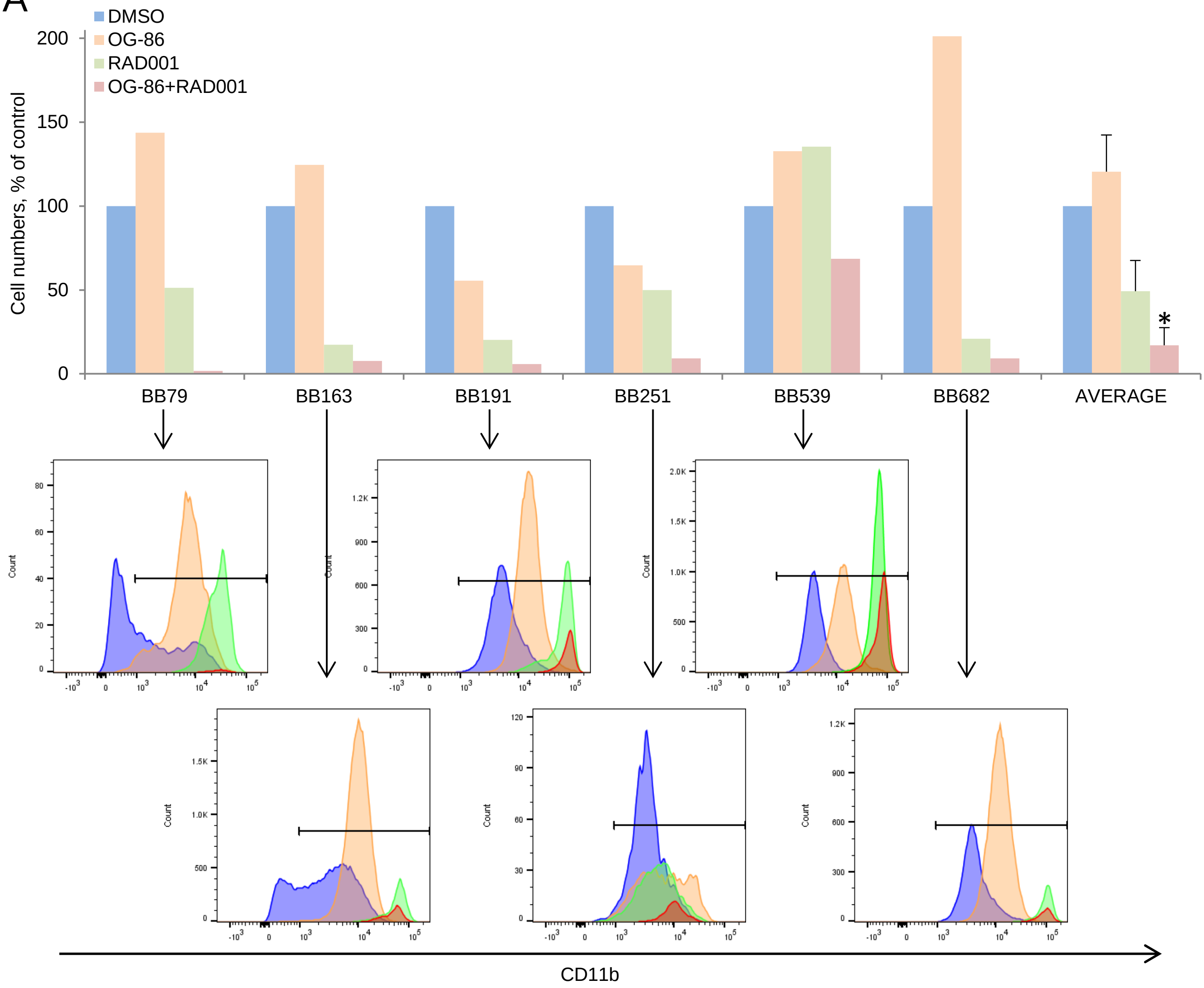

B

Biobank number	Karyotype
79	47,XX,der(3)t(3;9;14;4)(q2?1;q34;q24;q21),der(4)t(3;9;14;4),del(7)(q22q32),add(8)(q24),der(9)del(9)(p2?3)t(3;9;14;4),der(14)t(3;9;14;4),+18,add(19)(q13.3)x2[9]/46,XX[1]
163	45,X,-Y,t(8;21)(q22;q22)[8]/46,XY[2]
191	46,XX [20] with FLT3-ITD & mutant NPM1
251	46,XY,t(6;9)(p22;q34)[9]/46,XY,der(6)t(6;9),der(9)t(6;9)del(9)(q21q34)[2]
539	46,XY [20] with mutant NPM1
682	41~43,X,-Y,add(2)(q37),del(3)(q26),-4,del(5)(q?14),t(6;17)(p21;q21),der(7)t(4;7)(q2?4;q11.2), del(12)(p12p13),-18 [10]

Figure S8. Stromal co-culture experiments with primary AML cells.

Primary patient AML cells were thawed, recovered in stromal co-culture for 10 days and then treated with DMSO vehicle or the indicated compounds in stromal co-culture for seven days. (A) Relative cell numbers (upper panel) and relative expression of CD11b (lower panels) after a further seven days of culture are shown. (B) Table shows genotypes of primary AML samples evaluated. * indicates P<0.05 by one-way ANOVA with Fisher's least significant difference post hoc test for comparison of OG-86/RAD001 condition with all others.

Supplementary Materials and Methods

Human tissue, cell lines & cell culture

Human THP1 cells were from DMSZ (Braunschweig, Germany) and were cultured under standard conditions (5% CO_2, 37°C) in RPMI1640 supplemented with 10% FBS, 10U/mL penicillin, 10µg/mL streptomycin and 2mM L-glutamine (all from Life Technologies, Carlsbad, CA), or methylcellulose (H4320, Stem Cell Technologies, Vancouver, Canada). Cells were verified for authenticity by STR analysis and confirmed to be free from mycoplasma contamination. Culture densities were $5x10^4$ - $5x10^5$ for cells in liquid culture and the starting density was 10^3/ml in semi-solid culture. Colonies were enumerated after 5-7 days.

Cryopreserved leukaemic blast cells collected from the bone marrow or blood of patients at presentation were thawed and co-cultured on MS5 stromal cells, as described,[S1] in α-MEM medium supplemented with 12.5% heat-inactivated FBS, 12.5% heat-inactivated horse serum, 2mM L-glutamine, 57.2µM β-mercaptoethanol, 1µM hydrocortisone (Sigma Aldrich, St. Louis, MO) and IL-3, G-CSF and TPO (all at 20ng/ml; Peprotech, Rocky Hill, NJ) for seven days. This was to allow for recovery from cryopreservation. Cells were then transferred to fresh stromal layers and cultured for a further seven days in OG-86 250nM, RAD001 2µM or DMSO vehicle. Leukaemic cells (single cells) were readily separated from stromal cells (adhesive clumps) through disruption of the stromal layer by pipetting and then filtering the whole through a 75µm filter basket (Partec, Gorlitz, Germany).

Reagents

Inhibitors used were: MK2206 (#A10003), PP242 (#A10746), CCI779 (#A10906), BEZ235 (#HY-50673), AZD2014 (#A11303) (all from Generon, Slough, UK) and RAD001 (#07741, Sigma Aldrich). OG-86 was synthesised in house, as described.[4] The water-soluble compound OG-98 was obtained from Oryzon Genomics S.A.[S2,S3] Resorufin (alamarBlue) was from Invitrogen (Carlsbad, CA).

Antibodies, protein extraction and western blotting

For western blotting cells were lysed in a high salt lysis buffer (45mM HEPES (pH7.5), 400mM NaCl, 1mM EDTA, 10% glycerol, 0.5% Nonidet P-40, 6.25mM NaF, 20mM β-glycerophosphate, 1mM DTT, 20mM sodium orthovanadate and 1x Protease Inhibitor Cocktail (Roche)) and equal amounts of protein were loaded and separated by SDS-PAGE. Horseradish peroxidase-linked secondary antibodies (GE Healthcare, Little Chalfont, UK) and ECL (enhanced chemiluminescence; GE Healthcare) or SuperSignal (Pierce, Rockford, IL, USA) were used to detect immune complexes. Antibodies for western blotting were: ACTB (MAB1501; Millipore), RRAGA (#4357), MLST8 (#3274), phospho-p70 S6 Kinase (#9234), p70 S6 Kinase (#2708), phospho-p44/42 MAPK (#4370), p44/42 MAPK (#4695), phospho-4E-BP1 (#2855), 4E-BP1 (#9644) (all from Cell Signaling Technology; Danvers, MA, USA). All antibodies were used at a dilution of 1:1000 except ACTB (1:10,000).

Flow cytometry

Flow cytometry analyses were performed using either an LSR Model II BD FACSArray (BD Biosciences, Oxford, UK) or a Novocyte (Acea Biosciences, San Diego, CA, USA) flow cytometer. Cell-sorting experiments were performed using either Influx or FACSAria fluorescence-activated cell sorters (both from BD Biosciences). Antibodies used were anti-human CD11b-PE, anti-human CD14-FITC (fluorescein isothiocyanate), anti-human CD86-PerCP-eFluor710, and anti-Human CD117-PE (all eBioscience, Hatfield, UK). Apoptosis was assessed using a BD Pharmingen APC Annexin V kit (Oxford, UK), according to the manufacturer's instructions. Propidium iodide cell cycle analyses were performed as described.[4]

LSD1 and MAO biochemical assays

To determine the inhibitory activity of OG-98 versus LSD1, human recombinant LSD1 enzyme (#50100, BPS Bioscience, Abingdon, UK) was incubated for 15 minutes with OG-98 on ice in the assay buffer (50 mM sodium phosphate pH 7.4). The enzymatic reaction was initiated by the addition of KM dimethyl-H3K4 peptide substrate (#63677, Anaspec, Fremont, CA). After 30 min at 37°C, Amplex Red reagent and horseradish peroxidase solution were added according to manufacturer's instructions (Invitrogen) and left to incubate at room temperature in the dark. Conversion of the Amplex Red reagent to the fluorescent resorufin (λex = 540 nm, λem = 590 nm) was measured after 5 minutes using an Infinite F200 Tecan microplate reader (Tecan, Mannedorf, Switzerland). Kinetic parameters of LSD1 inhibition were assessed similarly, monitoring the formation of resofurin over time. To determine the inhibitory activity of OG-98 versus MAOA and MAOB, recombinant enzymes (#M7316 and # M7441 respectively, Sigma Aldrich) were incubated for 15 minutes with OG-98 in reaction buffer (100 mM Hepes pH 7.5). The enzymatic reaction was initiated by the addition of K_M kynuramine and incubated for 1 hour at 37°C and stopped by adding NaOH 2N (v/v). The conversion of kynuramine to 4-hydroxyquinoline was measured by fluorescence (λex = 320 nm, λem = 360 nm) using an Infinite F200 Tecan microplate reader. Kinetic parameters of MAOA and MAOB inhibition were assessed similarly monitoring the formation of 4-hydroxyquinoline over time. Fluorescence signals were background corrected and the IC50 value was calculated with GraphPad Prism Software. K_i and K_{inact} were calculated using the equations previously described for tranylcypromine.[S4]

Murine experiments

Cryopreserved leukaemic blast cells from a patient with a t(10;11) MLL gene rearrangement (BB518) were thawed and subjected to human $CD3^+$ immunomagnetic bead depletion (#130-050-101, Miltenyi Biotec, Bergisch Gladbach, Germany) to remove residual T-cells using the POSSELD program of an AutoMACS Pro device (Miltenyi Biotec), according to the manufacturer's instructions. Cells were placed overnight in StemSpan™ (Stem Cell Technologies) at a density of $2.5x10^4$/ml supplemented with 100ng/ml each of FL, IL-6, TPO and SCF (Peprotech) to allow for cell recovery from cryopreservation. $10x10^6$ million cells were then injected into sub-lethally irradiated (150cGy) NOD-SCID $IL2R\gamma^{-/-}$ (NSG) mice (female, aged 6–12 weeks; Envigo, Shardlow, UK) via the tail vein. Ten

weeks later mice were euthanized and engrafted human AML cells in BM and spleen cryopreserved. To establish four cohorts of primary cell xenoengrafted mice for drug treatment experiments (n=7 per cohort), sub-lethally irradiated (100cGy) mice were injected via the tail vein with $1x10^6$ $CD45^+$ human AML BB518 cells cryopreserved after the first round of xenotransplantation. Fourteen weeks later, when circulating human AML cells could be detected in a majority of animals, mice were allocated to four groups balanced for human $CD45^+$ blood chimerism and treated via oral gavage with either vehicle (H2O), RAD001 (5mg/kg), OG-98 (3mg/kg), or a combination of RAD001 (5mg/kg) and OG-98 (3mg/kg) for five days. At termination of the experiment, bone marrow cells were harvested from the long bones by crushing with a mortar and pestle. An oral dose of 5mg/kg RAD001 is expected to deliver a maximum concentration of ~4,500ng/mL one hour following administration, with a $t_{1/2}$ of 4.3hrs.[S5]

To model minimal residual human AML, we transplanted leukaemic blast cells from patient BB518 into NSG mice (female, aged 6–12 weeks; Envigo, Shardlow, UK) via the tail vein ($1x10^6$ per mouse). Prior to injection, cells were expanded *in vitro* in stromal co-culture (as described above) for 14 days. Three weeks after injection, mice were randomized into four groups (five mice per cohort) and treated via oral gavage with either vehicle (H_2O), RAD001 (5mg/kg), OG-98 (3mg/kg), or a combination of RAD001 (5mg/kg) and OG-98 (3mg/kg) for four weeks (five days treatment, two days off).

CRISPR screening

Human GeCKOv2 half libraries A and B in lentiCRISPRv2 (Addgene, Cambridge, MA) were amplified and maxi-prepped as described.[10] Details of verification of library representation, lentivirus manufacture and CRISPR screening procedures are in the Supplemental Information. Next, $2x10^8$ THP1 AML cells were infected with lentiviral particles (8µg/mL Polybrene; Millipore, Billerica, MA) at a multiplicity of infection of 0.3 (see Supplemental Information for details) and selected with 3ug/ml puromycin for seven days. $6{\times}10^7$ cells (representing 500-fold coverage of the library) were then washed with PBS and frozen for subsequent genomic DNA (gDNA) isolation. This was the early time point (Day 0). The remaining cells were treated with either DMSO or 250nM OG-86 for 18 days (sufficient for ten population doublings). Cells were divided between two groups of ten T225 flasks; each flask was seeded at $2{\times}10^5$ cells/mL in a total volume of 120mL. Cells were split every three days and medium and compound replaced. At the end of the experiment cells were frozen at -80°C for subsequent genomic DNA isolation using a QIAamp Blood Maxi kit (Qiagen, Hilden, Germany) as per manufacturer's instructions.

The sgRNA cassettes were amplified from genomic DNA and sequenced as detailed in the Supplemental Information. Sequencing reads were de-multiplexed using forward primer barcodes. Reads were trimmed using cutadapt (v1.7.1). The 20bp sgRNA sequences were then aligned to the GeCKO library using Bowtie (v1.0.1) allowing for up to one mismatched base. Mapped sgRNAs were filtered according to the following criteria before quantification: (i) any sgRNAs with other targets in the genome that match exactly or differ by only one base were discarded and (ii) for each biological

sample, any sgRNA with only a single read was removed. Normalised abundance for each sgRNA in a given sample was calculated as follows:

$$\text{Normalised reads per sgRNA} = (\text{reads per sgRNA/total reads for all sgRNAs in the sample}) \times 10^6$$

To identify significantly depleted sgRNAs and genes in the screen we used the Model-based Analysis of Genome-wide CRISPR/Cas9 Knockout (MAGeCK) method.[11]

Verification of uniform representation of CRISPR library

To verify uniform representation of sgRNAs a two-step PCR was performed in which PCR1 amplifies the lentiviral sequence containing the 20bp sgRNA cassette and PCR2 attaches Illumina sequencing adapters and barcodes. In PCR#1, 50ng of each half library was amplified using V2 adaptor primers (http://genome-engineering.org/gecko/) using Phusion High Fidelity master mix (Thermo). The thermocycling conditions were: 98C for 3min, 15 cycles of (98C for 20s, 60C for 20s, 72C for 30s) and 72C for 7min. For each half library there were two PCR#1 replicates. 5uL of each #PCR1 product was run in 1.5% TAE-agarose ethidium bromide gel to verify amplicon length (~300bp). For the PCR#2 reaction, which is carried out to add appropriate Illumina sequencing adapters to the PCR#1 product, 5uL of each PCR#1 product was used as template. Unique barcoded forward and reverse primer combination were used for each PCR#2 reaction as described.[12] Thermocycling conditions and cycle numbers were the same as for PCR#1. 5uL of PCR#2 product was run on a 1.5% TAE-agarose ethidium bromide gel to verify amplicon length (~350bp). All PCR#2 products were then pooled and gel purified from 2% TAE-agarose ethidium bromide gel using a QiaQuick kit, as per manufacturer's instructions (Qiagen, Hilden, Germany). The pooled library was then quantified by Qubit fluorometric quantitation (Thermo Fisher, Waltham, MA) and sequenced with 10% PhiX using a MiSeq System (150 cycles, v3 chemistry) (Illumina, San Diego, CA).

The sgRNA cassettes recovered from lentivirally-infected cells were amplified using PCR#1 and PCR#2 reactions as described above, but with some modifications. To preserve full library complexity and representation during sequencing, 244µg genomic DNA (~300-fold coverage) was used in each PCR#1 reaction per sample. For each PCR#1, we used 5ug gDNA using V2 adaptor primers and Phusion High Fidelity master mix (Thermo Fisher). The thermocycling conditions were: 98C for 30s, 19 cycles of (98C for 1s, 62C for 5s, 72C for 35s), 72C for 1min. 50 PCR#1 reactions were carried out per sample to capture the full representation of the screen. All the PCR#1 reactions for each biological sample were pooled together and 10uL of pooled #PCR1 product was used as template for PCR#2 reactions using barcoded primers for Illumina sequencing. The thermocycling conditions were: 98C for 30s, 19 cycles of (98C for 1s, 70C for 5s, and 72C for 35s), 72C for 1 min. For each sample, we performed at least 15 PCR#2 reactions (1 per 10K constructs). We included PCR#2 technical replicates for every sample (barcoded differently) to avoid duplicate sequences due to PCR over-amplification of constructs during sequencing. PCR#2 products of each technical replicate were pooled together and gel purified. The gel purified products were quantified by Qubit fluorometric quantitation (Thermo Fisher). Pooled PCR#2 products for each biological sample were

then normalized before combining uniquely barcoded separate PCR replicates for all biological samples. The amount of amplifiable DNA was assessed by real time PCR using P5 and P7 Illumina primers. High throughput sequencing was performed with 15% PhiX and a HiSeq 2500 system.

Generation of lentivirus for CRISPR screening & determination of MOI

For lentivirus production, both hGeCKO v2 half libraries were combined in an equimolar ratio. HEK293FT cells at 80-90% confluence in 20 T225 flasks in D10 were transfected with the combined library using polyethylenimine (PEI). For each flask 16µg hGeCKO v2 library (A+B), 8µg pCMVΔ8.91 (containing HIV gag, pol, rev and tat sequences) and 4µg pMD2.G (containing sequence for vesicular stomatitis virus envelope glycoprotein) were mixed with 84µL PEI. The solution was pulse vortexed and incubated at room temperature for 30min prior to dropwise addition in 40mL D10 to each flask. After overnight incubation the medium was replaced and 24hr later viral particles were harvested and stored at 4C; this step was repeated to generate a second viral harvest which was mixed with the first. Supernatant was centrifuged at 3,000 rpm at 4°C for 10 minutes and filtered through a 0.45µm low protein-binding membrane (Millipore). Lentiviral particles were concentrated as follows: 30% (w/v) polyethylene glycol (Bioultra, 6000) (PEG) (Sigma Aldrich) was prepared in 0.5M NaCl and the solution autoclaved and stored at 4°C. 20mL ice-cold 30% PEG was added to 20mL viral supernatant and incubated overnight at 4°C on a roller shaker. The virus-PEG mixture was then centrifuged at 3000g for 15min at 4°C. The supernatant was discarded and the pellet re-suspended in 1mL R10 per 50mL centrifuge tube. Concentrated virus was stored for later use at -80C.

To determine the viral titre of concentrated lentivirus stocks, 3×10^6 THP1 AML cells per well of a 12-well plate were spinoculated (2000rpm for 30min at 37°C) with varying dilutions of virus in R10 with 8µg/mL Polybrene (Sigma Aldrich). Next day, each well was divided between two wells of a 6-well plate and 3ug/ml puromycin was added to one of the replicate wells. After 72hr cells were counted using Trypan Blue dye exclusion and a haemocytometer. The viral dilution that resulted in 20-30% cell survival following puromycin selection in library transduced cells was used for transduction during the screen, and corresponds to a multiplicity of infection (MOI) of 0.2-0.3, assuming all infection events occur independently. At 20% cell survival following puromycin treatment, the expected percentage of cells infected by a single viral particle is estimated to be ~89%.

Lentiviral vectors for knockdown experiments

Lentiviral constructs (pLKO.1) used in knockdown experiments were from Sigma Aldrich: *RRAGA* #1 (TRCN0000291690), *RRAGA* #2 (TRCN0000291754), *MLST8* #1 (TRCN0000039761), *MLST8* #2 (TRCN0000039760), non-targeting control (NTC) (SHC002).

RNA sequencing and data analysis

Total RNA was extracted from AML cells using QIAshredder spin columns and an RNeasy Plus Micro Kit (Qiagen). RNA quality was confirmed using an Agilent Bioanalyzer (Agilent Technologies, Santa Clara, CA). Indexed PolyA libraries were prepared using either an Agilent SureSelect Strand Specific RNA Library Prep Kit (Agilent, Santa Clara, CA) (THP1 cells, 200ng total RNA) or a QuantSeq 3'

mRNA-Seq Library Prep Kit for Illumina Sequencing (Lexogen, Greenland, NH) (primary patient cells, 10ng total RNA). Libraries were quantified by quantitative PCR using a Kapa Library Quantification Kit for Illumina sequencing platforms (Roche, Basel, Switzerland). Paired-end (THP1 cells) or single end (primary cells) 75bp sequencing was carried out by clustering 1.8pM of the pooled libraries on a NextSeq500 sequencer (Illumina, San Diego, CA). Reads were mapped to the human genome (GRCh38 Gencode release 25) with STAR version 2.4.2a [S6] with the settings --outFilterMultimapNmax 20, --outFilterType BySJout, --alignSJoverhangMin 8 and --quantMode GeneCounts using the matching feature annotation GTF file for GRCh38 Gencode release 25. The number of uniquely mapped reads was 30-40 million. Differential gene expression analysis was performed using DESeq2 version 1.20.0.[S7] RPKM or FPKM (Reads or Fragments Per Kilobase of transcript per Million mapped reads) normalized values were also calculated using the DESeq2 package. Pre-ranked gene set enrichment analysis was performed with GSEA v2.0.14 software from www.broadinstitute.org/gsea7. Genes were rank ordered according to log2 fold change in expression.

Cytospin analyses

2-5x10^4 cells were suspended in 150µl PBS and, through centrifugation at 600xg for 5 minutes, were spun onto a microscope glass slide and left to air dry. Cells were fixed by incubation in methanol for 10 minutes and then stained by May-Grunwald (Sigma Aldrich; diluted 1:1 with Sorenson's Buffer (33.3mM KH_2PO_4, 64.75mM Na_2HPO_4, pH 6.8)) staining for 20 minutes and then Giemsa (Sigma Aldrich; 10x diluted with Sorenson's Buffer) staining for 30 minutes. Stained slides were washed under running tap water and left in Sorenson's buffer for five minutes prior to one final brief wash with tap water. Slides were left to air dry before cells were permanently mounted with a coverslip and DPX neutral mounting media (VWR, Radnor, PA). Images were obtained using a Leica SCN400 histology scanner (Leica, Solms, Germany).

alamarBlue assay

2500 cells were plated into each well of a 96-well plate and incubated with inhibitors or DMSO vehicle for 5 days. 20µl alamarBlue was added to the cells and incubated for 4h at 37°C. Resorufin fluorescence was measured using a fluorescence-based plate reader (POLARstar Omega, BMG Labtech, Aylesbury, UK).

Amino acid depletion assay

Amino acid-free RPMI medium powder (R8999-04A, US Biological, Salem, MA) was complemented with 2g/L sodium bicarbonate and 0.8g/L sodium phosphate, dissolved in water, adjusted to pH7.4 and sterile filtered. Complete RPMI containing a 1x concentrated solution of amino acids was obtained by complementing amino acid-free RPMI medium with RPMI 1640 amino acids solution (R7131, Sigma Aldrich), adjusted to pH7.4 and filtered. 10U/mL penicillin, 10µg/mL streptomycin, 2mM L-glutamine were added shortly before use. Amino acid depletion media were obtained by supplementing the amino acid free RPMI with individual amino acids (all from Sigma Aldrich) as follows: L-Arginine (0.2mg/ml), L-Asparagine (0.05mg/ml), L-Aspartic acid (0.02mg/ml), L-

Cysteine.2HCl (0.0652mg/ml), L-Glutamic acid (0.02mg/ml), L-glutamine (0.3mg/ml), Glycine (0.01mg/ml), L-Histidine (0.015mg/ml), Hydroxy-L-Proline (0.02mg/ml), L-Isoleucine (0.05mg/ml), L-Leucine (0.05mg/ml), L-Lysine.HCl (0.04mg/ml), L-Methionine (0.015mg/ml), L-Phenylalanine (0.015mg/ml), L-Proline (0.02mg/ml), L-Serine (0.03mg/ml), L-Threonine (0.02mg/ml), L-Tryptophan (0.005mg/ml), L-Tyrosine.2Na_2H_2O (0.03mg/ml), L-Valine (0.02mg/ml). 2500 cells per well of a 96 well plate were incubated with OG-86 250nM in the indicated media. Up regulation of cell surface markers such as CD86 was assessed after two days by flow cytometry. Viability was assessed after 5 days using an alamarBlue assay.

References

S1. van Gosliga D, Schepers H, Rizo A, van der Kolk D, Vellenga E, Schuringa JJ. Establishing long-term cultures with self-renewing acute myeloid leukemia stem/progenitor cells. Experimental Hematology 2007;35:1538-49.

S2. Guibourt N, Ortega Muñoz A and Castro-Palomino Laria JC. Phenylcyclopropylamine derivatives and their medical use. Patent WO2010/084160, filed January 21, 2010, and published July 29, 2010.

S3. Guibourt N, Ortega Muñoz A and Castro-Palomino Laria JC. Oxidase inhibitors and their use. Patent WO 2010/043721, filed October 17, 2008, and published April 22, 2010.

S4. Yang M, Culhane JC, Szewczuk LM, Gocke CB, Brautigam CA, Tomchick DR et al. Structural basis of histone demethylation by LSD1 revealed by suicide inactivation. Nature Structural and Molecular Biology 2007;14:535–539.

S5. O'Reilly T, McSheehy PM, Kawai R, Kretz O, McMahon L, Brueggen J et al. Comparative pharmacokinetics of RAD001 (everolimus) in normal and tumor-bearing rodents. Cancer Chemotherapy and Pharmacology 2010;65:625-639.

S6. Dobin A, Davis CA, Schlesinger F, Drenkow J, Zaleski C, Jha S, et al. STAR: ultrafast universal RNA-seq aligner. Bioinformatics 2013;29:15-21.

S7. Love MI, Huber W, Anders S. Moderated estimation of fold change and dispersion for RNA-seq data with DESeq2. Genome Biology 2014;15:550.
